# Central Auditory Processing Disorder in Patients with Amnestic Mild Cognitive Impairment

**DOI:** 10.1155/2022/9001662

**Published:** 2022-12-15

**Authors:** Ga-Young Kim, HyangHee Kim, Hee Jin Kim, Sang Won Seo, Duk L. Na, Chung Mo Nam, Byoung Seok Ye, Il Joon Moon

**Affiliations:** ^1^Graduate Program in Speech and Language Pathology, Yonsei University, Seodaemun-gu, Seoul 03722, Republic of Korea; ^2^Hearing Research Laboratory, Samsung Medical Center, Gangnam-gu Seoul 06351, Republic of Korea; ^3^Medical Research Institute, Sungkyunkwan University School of Medicine, Suwon, Republic of Korea; ^4^Department and Institute of Rehabilitation Medicine, Yonsei University College of Medicine, Seodaemun-gu, Seoul 03722, Republic of Korea; ^5^Department of Neurology, Samsung Medical Center, Sungkyunkwan University School of Medicine, Gangnam-gu, Seoul 06351, Republic of Korea; ^6^Alzheimer's Disease Convergence Research Center, Samsung Medical Center, Gangnam-gu, Seoul 06351, Republic of Korea; ^7^Department of Biostatistics and Computing, Yonsei University College of Medicine, Seodaemun-gu, Seoul 03722, Republic of Korea; ^8^Department of Neurology, Yonsei University College of Medicine, Seodaemun-gu, Seoul 03722, Republic of Korea; ^9^Department of Otorhinolaryngology-Head and Neck Surgery, Sungkyunkwan University School of Medicine, Samsung Medical Center, Gangnam-gu, Seoul 06351, Republic of Korea

## Abstract

**Background:**

This study was conducted to comprehensively examine the central auditory processing (CAP) abilities of patients with amnestic mild cognitive impairment (aMCI) as well as to compare the results with cognitively normal elderly controls.

**Methods:**

A total of 78 participants were screened through pure-tone audiometry and word recognition score in order to exclude peripheral auditory dysfunction. Forty-five people passed screening tests, and 33 people failed. Finally, 25 aMCI (mean age = 71.52 ± 4.8; male : female = 24 : 76) and 20 controls (mean age = 73.45 ± 4.32; male : female = 45 : 55) were enrolled in the study. Seven CAP tests (frequency pattern test, duration pattern test, Gap-In-Noise^©^ test, dichotic digits test, low-pass filtered word test, speech perception in noise test, and binaural fusion test) were conducted only after the two groups passed the screening. A linear mixed model was applied to analyze CAP tests except for the binaural fusion test. For the binaural fusion test, the independent *t*-test was used to compare the means of test score between two groups.

**Results:**

The aMCI group had a decrease in the mean score of the frequency pattern test, duration pattern test, Gaps-In-Noise^©^ test, dichotic digits test, and speech perception in noise test compared with the control group.

**Conclusion:**

The aMCI group's CAP abilities were significantly lower than those of the control group. Thus, if the cognitive assessment and hearing evaluation are conducted in combination, the sensitivity of the diagnostic process for aMCI will be increased.

## 1. Introduction

Central auditory processing (CAP) is the perceptual processing of auditory information within the central auditory nervous system (CANS) [1]. CAP consists of mechanisms that serve to preserve, refine, analyze, modify, organize, and interpret information from the auditory periphery. These mechanisms underline the following skills, including temporal processing, auditory discrimination, dichotic listening, monaural low-redundancy and binaural processing [1].

Central auditory processing disorder (CAPD) is defined as a deficit in terms of neural processing of auditory information in the central nervous system [1]. CAPD may lead to or be asscoiated with difficulties faced in the context of higher order language, communication, and learning. CAPD may also coincide with other disorders (e.g., attention-deficit/hyperactivity disorder, language impairment, and learning disability) [1]. CAPD is a term referring to a functional disorder, not a single disease.

A comprehensive assessment of the lesion and function of the CANS should include behavioral tests in at least five areas [1]. The five behavioral test areas are auditory temporal processing, auditory discrimination, dichotic listening, monaural low-redundancy, and binaural interaction test [1]. Auditory temporal processing is an ability to perceive a sound or the alteration of sound within a limited or defined the domain [2]. There are four subcomponents of temporal processing of auditory signals including temporal ordering, temporal discrimination, temporal integration, and temporal masking. Temporal ordering plays an important role in speech perception with the ability to process two or more stimuli in time order [3]. Frequency pattern test (FPT) and duration pattern test (DPT) are most widely used to measure temporal ordering. These two pattern tests are sensitive to hemispheric lesions as well as interhemispheric dysfunction [4].

The auditory system is required to discriminate small timing differences when processing speech. Temporal discrimination is defined as the shortest duration of time in which an individual can discriminate between two auditory signals [2]. A common method used to assess temporal discrimination is to establish a gap detection threshold (GDT). GDT task requires participants to response whenever they hear a “silent” interval embedded in an ongoing noise burst [2]. The Gaps-In-Noise (GIN^©^) test is an example of GDT task.

Dichotic listening refers to the ability to integrate or separate different acoustic stimuli that are simultaneously provided to each ear and assess the cerebral hemisphere connectivity in the central auditory region [5]. Dichotic listening is divided into various tests depending on the type of stimulus used, such as dichotic digits, dichotic consonant vowels, Staggered Spondaic Words, and dichotic sentences. Of these, the dichotic digits test (DDT) is easier to measure the CAP of patient with cognitive impairment because it is less affected by working memory than other dichotic listening tests [13].

The monaural low-redundancy test evaluates the CAP system by presenting stimuli that lower the redundancy inherent in acoustic signals through frequency filtering, temporal compression, or noise presentation in the unilateral ear. The low-pass filtered word (LPFW) test is one of the low-redundancy tests that can assess auditory closure by reducing spectral aspects of speech through frequency filtering [6]. Auditory closure means the capability of the normal listener to make use of intrinsic and extrinsic redundancies to cover missing or inaccurate parts of the auditory stimulus and understand the whole message. When speech is degraded (e.g., extrinsic redundancy is reduced), listener with reduced intrinsic redundancy (due to CANS dysfunction) demonstrates a significant decline in speech recognition performance [7].

Mild cognitive impairment is interim phase between normal cognitive and Alzheimer's disease (AD) or another type of dementia [8]. Mild cognitive impairment (MCI) is a condition in which cognitive decline is outside the normal range but not severe enough to be diagnosed as dementia. In particular, MCI with memory deficits defined as amnestic MCI (aMCI) is considered a prodromal state of AD. The clinical diagnostic criteria for aMCI are as follows: (1) memory complaint, preferably corroborated by an informant; (2) essentially normal general condition; (3) largely normal activities of daily living; (4) objective memory impairment for age; and (5) not demented [8].

Both peripheral and CAP dysfunctions have possible influence on late-life cognitive disorders [9]. However, most previous research has focused on the association between peripheral hearing loss and cognition [10, 11], and little has been explored about CAP performances in patients with aMCI. Furthermore, claims have been made that cognitive decline was more related to CAPD than peripheral hearing loss [12], and there were no differences in peripheral hearing acuity between aMCI and normal cognitive groups [13–16].

Few studies have shown that CAPD seemed to be frequent in patients with aMCI [13–16]. One study demonstrated that subjects with probable aMCI performed worse on temporal processing and competing acoustic signals [14]. However, subjects of this study were divided into community-dwelling elderly with and without probable aMCI according to the screening test. There is a need to replicate findings in a clinically defined population with through neuropsychological evaluation. Another study confirmed that DDT is more decisive in the AD group than the aMCI group [13]. This implies CAPD is apparent in aMCI; however, CAP needs to be addressed in multiple domains. More investigations are necessary to identify multifactorial relationships between CAP and aMCI.

The purpose of this study was to comprehensively examine the CAP abilities of patients with aMCI. Furthermore, the results obtained were compared with the cognitively normal elderly control to reveal CAP characteristics in patients with aMCI.

## 2. Methods

### 2.1. Participants

All participants visited the outpatient clinic of the Department of Neurology at Samsung Medical Center (SMC) in Seoul from March to December 2020.

The patients with aMCI (aMCI group) were diagnosed by a neurologist based on Petersen's criteria [8]: (1) subjective memory complaint reported by the patient of informants; (2) normal activities of daily living; (3) normal general cognitive function; (4) objective memory complaint as defined by score less than 16 percentile on memory domain of neuropsychological test; and (5) no dementia.

The cognitively normal control group (control group) met these criteria: (1) no significant underlying medical, neurologic, or psychiatric illness; and (2) *z* scores of each of the five cognitive domains (attention, language, visuospatial function, memory, and frontal-executive function) of the Seoul Neuropsychological Screening Battery (SNSB) were −1.0 or above.

The two groups also had to meet these criteria for hearing acuity: (1) no conductive hearing loss on pure-tone audiometry; (2) hearing threshold levels of ≤40 dB HL at 0.5, 1, 2, and 4 kHz in each ear; (3) no greater than 10 dB HL of inter-aural asymmetry on pure-tone average; and (4) a word recognition score (WRS) ≥80% for each ear.

A total of 78 participants were screened through pure-tone audiometry and WRS for inclusion in the study. Forty-five participants passed the screening tests, and 33 participants failed. Finally, 25 aMCI and 20 control were enrolled in the study. The characteristics of participants were shown in Table 1.

### 2.2. Ethical Consideration

This study was approved by the institutional review board (IRB) at SMC, Seoul, South Korea, in accordance with the Declaration of Helsinki (IRB file no. 2020-01-114).

### 2.3. Procedure

#### 2.3.1. Audiometric Assessments (Screening Assessments)

The pure-tone audiometry was performed with standard audiologic procedures. Air and bone conduction thresholds were measured with a clinical pure-tone audiometry (GSI 61; Grason-Stadler, Eden Prairie, MN, USA) in a double-walled soundproof booth. Pure-tone average at 0.5, 1, 2, and 4 kHz for each ear was calculated.

For the WRS, fifty monosyllabic words from the Korean standard-monosyllabic word list [17] were presented at the most comfortable level (MCL) in each ear through the TDH-39 headphone. The MCL was determined to indicate the patient when the speech is perceived to be at a comfortable level. The participants were asked to repeat the word back to the tester. Percentage-correct scores were calculated for scoring.

#### 2.3.2. CAP Tests

A licensed audiologist (G.-Y.K.) performed the following five CAP tests.

*(1) Frequency Pattern Test*. A high-frequency pure tone of 1122 Hz and a low frequency pure tone of 880 Hz were used as the pattern stimulus. A pattern consisted of three 150 ms pure tones (e.g., low-low-high) and two 200 ms inter-tone intervals [4]. After connecting the CD player (YAMAHA TSX-B232; YAMAHA Corp., Hamamatsu, Shizuoka, Japan) to the audiometer, test items were presented at the MCL through a loudspeaker located 1 m from the participant. The participants responded to the patterns they heard in response to high and low sounds by labeling and humming. Percentage-correct scores were calculated for scoring.

*(2) Duration Pattern Test*. A pattern consisted of three 1000 Hz pure tones (e.g., long-long-short) and two 300 ms inter-tone intervals. The tones in each pattern were 250 and 500 ms, respectively [4]. After connecting the CD player to the audiometer, test items were presented at the MCL through a loudspeaker located 1 m from the participant. The participants responded to the patterns they heard in response to short and long sounds by labeling and humming. Percentage-correct scores were calculated for scoring.

*(3) GIN^©^ Test*. The test comprised a serial of 6-second segments of noise including 0–3 silent gaps per noise segment. The inter-stimulus interval between consecutive noise segments was 5 seconds, and the gap durations were 2, 3, 4, 5, 6, 8, 10, 12, 15 and 20 ms. Both gap duration and the location of gaps within the noise segments were pseudorandomized. Furthermore, the number of gaps per noise segment was diverse. After connecting the CD player to the audiometer, test items were presented at the MCL in each ear through the TDH-39 headphone. The participants were instructed to press the response button as soon as they heard a gap. Two measures were derived for each ear during the procedure. These include an approximated GDT and a combined percentage correct identification score across all gap durations. The approximate threshold (A.th.) was defined as the shortest gap duration for which there are at least “four of six” correct identifications [2].

*(4) Dichotic Digits Test*. The test stimuli consisted of two-digit pairs of numbers from 1 to 10 except for 2, which showed the most errors due to acoustic similarity [18]. After connecting the CD player to the audiometer, test items were presented at the MCL in two ears simultaneously through the TDH-39 headphone. The participants followed all four numbers regardless of the order of the number they heard. Percentage-correct scores were calculated for scoring.

*(5) Low-Pass Filtered Word Test*. A low frequency filtration of monosyllabic words at 1500 Hz was used [19]. After connecting the CD player to the audiometer, test items were presented at the MCL in each ear through the TDH-39 headphone. The participants were asked to repeat the word back to the tester. Percentage-correct scores were calculated for scoring.

*(6) Speech Perception in Noise Test*. The Korean Speech Intelligibility in Noise [20] with 4-talker babble noise was used. The noise levels were set to 0 and −5 dB SNR. According to the participants, the test lists and the noise levels are randomly presented. After the participant was seated in the middle of four speakers, sentences are presented from the front speaker, whereas noise is presented from four speakers at 45°, 135°, 225°, and 315°. The participants were asked to repeat the sentence back to the tester. Percentage-correct scores were calculated for scoring.

*(7) Binaural Fusion Test*. The test words were filtered with different segments of low pass (1200 Hz cutoff) and high pass (2100 Hz cutoff). After connecting the CD player to the audiometer, test items were presented at the MCL in each ear through the TDH-39 headphones. The test stimuli were presented as different segments of band-pass filtered speech to the two ears with a low-band-pass filtered speech stimulus presented to right ear and a high-band-pass filtered presentation of the same speech stimulus to left ear. The participants were asked to repeat the word back to the tester. Percentage-correct scores were calculated for scoring.

#### 2.3.3. Neuropsychological Assessments

The SNSB is a standardized neuropsychological assessment that evaluates five cognitive domains, which involves attention, language, visuospatial function, memory, and frontal-executive function [21]. Composite score on each of the domains was calculated. The *z* scores were calculated according to the normative data derived from age- and year of education-matched Korean population.

### 2.4. Statistical Analysis

Regarding demographic features, continuous variable was compared with independent sample *t*-test and categorical variables with chi-squared tests, appropriately.

A linear mixed model (LMM) was applied to analyze CAP tests except for the binaural fusion test (BFT). Regarding the FPT and DPT, two fixed effects were included: one dichotomous within-subjects predictor (response type) and one dichotomous between-subjects predictor (group). Possible differences in the group across response type were analyzed according to *response type∗group* interactions. With respect to the GIN^©^ test, DDT, and LPFW test, two fixed effects were included: one dichotomous within-subjects predictor (test ear) and one dichotomous between-subjects predictor (group). Possible differences in the group across test ear were analyzed according to *test ear∗group* interactions. LMM with an “unstructured covariance matrix” was used, that is, a covariance matrix upon which no constraints have been imposed. The parameter estimates for the fixed effects were analyzed, and the coefficient estimate, standard error (SE), *t*-value, and *p*-value were reported. For the BFT, the independent *t*-test was used to compare the means of test score between two groups.

All statistical analyses were performed with SPSS 25.0 (IBM Corp., Armonk, NY, USA).

## 3. Results

### 3.1. FPT

The response type (*p* = 0.036) and group (*p* < 0.001) significantly predicted the FPT score. The labeling response had a lower mean FPT score than the humming response (*β* ± SE: −20.50 ± 9.62). The aMCI group showed a lower performance than the control group (*β* ± SE: −37.43 ± 9.13). However, there was no interaction effect between response type and group (*p* = 0.851) (Table 2).

### 3.2. DPT

The group (*p* = 0.031) only significantly predicted the DPT score. The aMCI group had a lower mean DPT score than the control group (*β* ± SE: −14.27 ± 6.51). There was no interaction effect between response type and group (*p* = 0.065) (Table 3).

### 3.3. GIN^©^ Test

In the case of the GIN^©^ test, one subject was excluded from the analysis because of the lack of understanding on the test. The results of the GIN^©^ test were reported with two parameters: A.th. of gap detection and percentage correct.

For the A.th. of gap detection, the group (*p* = 0.001) only significantly predicted the A.th. of gap detection. The aMCI group had a longer mean threshold than the control group (*β* ± SE: 1.92 ± 0.55). There was no interaction effect between test ear and group (*p* = 0.309) (Table 4). For the percentage correct, the group (*p* = 0.009) only significantly predicted the GIN^©^ test score. The aMCI group had a lower mean score than the control group (*β* ± SE: −8.42 ± 3.15). There was no interaction effect between test ear and group (*p* = 0.447).

### 3.4. DDT

The group (*p* = 0.029) only significantly predicted the DDT score. The aMCI group had a lower mean DDT score than the control group (*β* ± SE: −18.20 ± 8.18). There was no interaction effect between test ear and group (*p* = 0.603) (Table 5).

### 3.5. LPFW Test

The test ear (*p* = 0.502) and group (*p* = 0.651) did not significantly predict the LPFW score. There was no interaction effect between test ear and group (*p* = 0.753).

### 3.6. SPIN Test

There were significant interactions between noise level and group (*p* < 0.001). The aMCI group had a lower mean Speech Perception in Noise (SPIN) test score than the control group, and the difference on the mean SPIN test score was greater in −5 dB SNR.

### 3.7. BFT

There was no significant difference in the BFT scores between the groups (*t* = −0.039, *p* = 0.969).

## 4. Discussion

In this study, comprehensive CAP behavioral tests were conducted for patients with aMCI, and the results obtained were compared with the control group. The aMCI group had a decrease in the mean score of the FPT, DPT, GIN^©^, DDT, and SPIN test compared with the control group. The results of each tests are discussed in more detail below.

Two temporal pattern tests were used to confirm temporal ordering ability. The aMCI group showed lower scores than the control group for both tests. Regarding response type, the labeling performance of the FPT was worse than that of the humming performance in the aMCI group. However, in the DPT, there were no differences between the labeling and humming performances in both groups. These results were consistent with previous studies in which the aMCI group had significant lower scores in pitch pattern sequence [16] and tone duration discrimination [22] compared with the control group.

Temporal ordering is the ability to process two or more auditory stimuli in sequence. Several perceptual and cognitive processes are required to accurately recognize, identify, and sequence auditory patterns [7]. For instance, to accurately report the sequence of tones, which consist of triads of pure tones of two different frequencies, the participants go through the following steps: (1) perceiving the tonal stimuli through the peripheral organs; (2) storing temporarily the perceived tonal stimuli in the working memory and decoding the frequency of each stimulus by comparing it with the frequency information stored in the long-term memory; and (3) arranging the tonal stimuli in order and verbally labeling them [23].

These auditory pattern perception and recognition processes are not limited to the cerebral hemisphere alone, but rather require integration of information from both hemispheres via the corpus callosum [7]. In other words, the right hemisphere recognizes the acoustic contour and reacts by humming, followed by transfer to the left hemisphere via the corpus callosum, and the left hemisphere verbally labels the acoustic contour (i.e., “high/low” or “long/short”).

Furthermore, the response observed in the temporal pattern test can also be used to explore the neurological correlates associated with it. If both the humming and labeling responses are declined, the right hemisphere's function is deteriorated. Rather, an individual who can hum but not verbally label tonal patterns most likely suffers from dysfunction in interhemispheric transfer to the left hemisphere, or dysfunction in the left hemisphere [7].

In the study, the aMCI group's labeling performance was worse than that of the control group. This phenomenon suggests that the aMCI group has a problem when transmitting information between the cerebral hemispheres or a problem with the linguistic markers of the left hemisphere rather than a problem in the acoustic contour resolution of the right hemisphere.

The GIN^©^ test was performed to identify the temporal discrimination or resolution [2], and consequently, the aMCI group had a longer A.th. of gap detection and a lower percentage correct than the control group [14, 15, 24]. The temporal discrimination or resolution refers to the shortest interval between two auditory signals. The temporal discrimination or resolution is commonly known as temporal auditory acuity or minimum integration time [7]. The reason that the aMCI group's ability to detect gaps was decreased compared with the control group was that cognitive function could affect the temporal discrimination or resolution. In this context, cognitive function refers to speed of processing, executive function, and auditory attention.

First, regarding the speed of processing, the A.th. of gap detection might have been longer because the overall speed of the processing slows as age increases and that most of the patients with cognitive impairment, such as aMCI, are elderly [14]. In one study, the mean of A.th. of gap detection of 100 normal-hearing young adults (18–31 years) was 4.19 ms [25].

In addition, recent research estimated the effect of executive function and auditory attention based on the GIN^©^ test with patients with aMCI [24]. The impulsivity index indicated executive function, and the index was calculated in two ways: “Impulsivity hits” were calculated by dividing Correct Hits or true positives by False Hits or false alarms, and “impulsivity total items” were computed by dividing the total items by False Hits or false alarms. If there was no stimulus presented and the participant responded, it was considered a false alarm. The impulsivity hits and the impulsivity total items imply that the index increases as the executive function decreases. The inattentiveness index represented auditory attention. The index was obtained by dividing the total number of items presented above an individual participant's measured threshold by the number of gaps that participant failed to identify, despite the fact that the duration of those gaps exceeded the participant's measured gap threshold. As the inattentiveness index increases, auditory attention declines. The aMCI group showed a higher impulsivity and inattentiveness index than the control group. In short, the lower performance in the GIN^©^ test by the aMCI group is due to poor executive function and auditory attention.

The definition of dichotic listening is the simultaneous stimulation of both ears, but with a different stimulus in each ear. As a result of the percentage correct for DDT according to the group and the test ear, the aMCI group showed poorer performance than the control [13, 16]. Dichotic listening gradually declines in performance during the transition from subjective memory impairment (SMI) to aMCI and even early AD [13]. A 5-year longitudinal study was conducted to determine the changing dichotic listening performance in patients with SMI, aMCI, and early AD [26]. Dichotic listening performance declined significantly in the group transitioning from SMI or aMCI to AD and in the AD group at the baseline.

As for the test ear, there was no significant difference between the aMCI and SMI groups regarding ear advantage (EA) [13]. The 5-year longitudinal study of DDT showed no significant difference between the aMCI and SMI groups in both ears [26]. This phenomenon can be interpreted as the neurological mechanism of dichotic listening. In the DDT, EA can be explained through handedness, the dominant language hemisphere, and the dominant ear [27]. The left hemisphere is the dominant hemisphere for language in most right-handed people, and the right hemisphere is the dominant hemisphere for language in most left-handed people. EA could be predicted by the dominant hemisphere for language in that the left hemisphere shows right ear advantage (REA) and the right hemisphere shows left ear advantage. The reason is that the signal is stronger in the contralateral ascending auditory pathway compared to the ipsilateral connection. For example, in DDT, the left hemisphere is responsible for the performance of the right ear.

In the study, most participants were right-handed, so the dominant hemisphere for language was predicted to be the left hemisphere. In other words, if the left hemisphere functions properly, the REA phenomenon is prominent. However, it is interpreted that the REA phenomenon disappeared as the REA score declined due to problems, such as overall cortical thinning in the left hemisphere. Many studies have reported left cortical thinning in the aMCI group [28, 29]. In addition, the temporal processing tests described above also suggested a left hemisphere lesion in the aMCI group.

Among the CAP abilities, speech perception in noise condition is one of the most prominent symptoms in patients with aMCI [14, 15, 30–32]. The aMCI group had a lower mean SPIN test score compared to the control group, and the difference in the mean SPIN test score was greater in the more challenging condition. One possible explanation for the results could be related to insufficient cognitive resources. Degraded auditory signals require greater cognitive resources for auditory perceptual processing and diversion from other cognitive tasks to effortful listening, eventually resulting in cognitive reserve depletion [33]. The aMCI group is unable to use enough cognitive resources in adverse listening situations, and due to that, the overall cognitive ability declines below that of the control group [33, 34]. This led to the postulation that the aMCI group has a poorer overall cognitive ability than the control group, making it difficult to employ cognitive resources adequately in adverse listening situations.

These results can be also explained using the Ease of Language Understanding (ELU) model [34, 35]. According to the ELU model, when input signals and phonological representations are inconsistent, working memory and/or frontal-executive function are involved in language processing, that is, the input signal compares rapidly, automatically, and multimodally phonological representations in semantic long-term memory through a phonological buffer, also known as a Rapid, Automatic, Multimodal Binding of PHOnology (RAMBPHO) buffer. If the input signal corresponds to phonological representations, then the input signal is understood without the need for working memory or frontal-executive function. However, additional working memory or frontal-executive function is required when the intelligibility of the input signal is degraded due to background noise. In the study, the aMCI group had a lower percentage correct under all conditions compared with the control group. This is believed to be due to the aMCI group having a lower frontal-executive function than the control group and, thus, could not use the RAMBPHO buffer efficiently. In one former study, each time a frontal-executive function SD score decreased by 1, the score of the speech perception in a noisy situation decreased by 9.2 percentage points [36].

This study confirmed that the aMCI group's CAP abilities were significantly lower than that of the control group. One goal of the current diagnostic practice of dementia is earlier diagnosis and timely, appropriate treatment to prevent or delay further deterioration. Patients with aMCI are more likely to develop dementia; thus, if the cognitive assessment and hearing evaluation are conducted in combination, the sensitivity of the diagnostic process for dementia will be increased. Furthermore, CAP tests can be used as a cost-effective screening tool for cognitive decline.

The current study has some limitations. First of which was a small sample size, meaning the generalizability of the findings to other patient populations is unclear. Another limitation was that only behavioral tests were performed. Further studies will be required to investigate both behavioral and electrophysiological tests, such as auditory brainstem response to fully understand the CAP abilities in patients with aMCI.

## Figures and Tables

**Table 1:** Characteristics of participants.

Variables	Categories	aMCI (*n* = 25),	Control (*n* = 20),	*χ* ^2^ or *t*	*p* valu*e*
		*N* (%) or mean (SD)	*N* (%) or mean (SD)		
Sex	Men	6 (24)	9 (45)	2.205	0.138
	Women	19 (76)	11 (55)		
Age, year		71.52 (4.80)	73.45 (4.32)	−1.401	0.169
Year of education		11.00 (4.50)	12.15 (4.89)	−0.820	0.417
Handedness	Right	23 (92)	19 (95)	0.161	0.688
	Left	2 (8)	1 (5)		
PTA^a^, dB HL	Right	21.25 (8.35)	23.31 (9.58)	−0.771	0.445
	Left	22.00 (8.32)	25.71 (9.36)	−1.405	0.167
WRS, %	Right	95.28 (4.28)	93.20 (6.10)	1.292	0.205
	Left	96.48 (4.21)	94.70 (3.51)	1.514	0.137
SNSB, *z* score	Attention	−0.29 (0.95)	0.23 (1.07)	−1.675	0.101
	Language	−0.65 (1.70)	0.48 (0.52)	−3.129	0.004∗∗
	Visuospatial	−1.78 (2.86)	0.33 (0.78)	−3.518	0.001∗∗
	Memory	−2.40 (1.37)	0.40 (0.73)	−8.636	<0.001∗∗∗
	Frontal-executive function	−1.49 (2.25)	0.23 (0.98)	−3.387	0.002∗∗
Amyloid PET	Negative	2 (8.7)	14 (73.7)	18.634	<0.001∗∗∗
	Positive	21 (91.3)	5 (26.3)		
APOE4 *ε*4 carrier	Non-carrier	7 (33.3)	14 (66.7)	6.702	0.010∗
	Carrier	16 (72.7)	6 (27.3)		

^a^The four-frequency average (0.5, 1, 2, and 4 kHz) for each ear was calculated.

Abbreviations: aMCI, amnestic mild cognitive impairment; PTA, pure-tone average; PET, positron emission tomography; SD, standard deviation; SNSB, Seoul Neuropsychologic Screening Batter; WRS, word recognition score. ∗*p* <0.05, ∗∗*p* <0.01, ∗∗∗*p* <0.001.

**Table 2:** Estimated fixed effects of predictors for frequency pattern test.

Fixed effects	Estimated coefficient (*β*)	S.E.	*t*	*p* value
Intercept	91.83	6.80	13.50	<0.001∗∗∗
Response type				
Labeling	−20.50	9.62	−2.13	0.036∗
Humming (reference)	0	0		
Group				
aMCI	−37.43	9.13	−4.10	<0.001∗∗∗
Control (reference)	0	0		
Response type∗ group	−2.43	12.91	−0.19	0.851

Abbreviations: aMCI, amnestic mild cognitive impairment; S.E., standard error. ∗*p* < 0.05, ∗∗∗*p* < 0.001.

**Table 3:** Estimated fixed effects of predictors for duration pattern test.

Fixed effects	Estimated coefficient (*β*)	S.E.	*t*	*p* valu*e*
Intercept	97.33	4.85	20.06	<0.001∗∗∗
Response type				
Labeling	−6.50	6.86	−0.95	0.346
Humming (reference)	0	0		
Group				
aMCI	−14.27	6.51	−2.19	0.031∗
Control (reference)	0	0		
Response type∗ group	−17.23	9.21	−1.87	0.065

Abbreviations: aMCI, amnestic mild cognitive impairment; S.E., standard error.. ∗*p* < 0.05, ∗∗∗*p* < 0.001.

**Table 4:** Estimated fixed effects of predictors for approximate threshold of gap detection.

Fixed effects	Estimated coefficient (*β*)	S.E.	*t*	*p* value
Intercept	7.75	0.41	18.95	<0.001∗∗∗
Test ear				
Right	−0.20	0.58	−0.35	0.730
Left (reference)	0	0		
Group				
aMCI	1.92	0.55	3.46	0.001∗∗
Control (reference)	0	0		
Test ear∗ group	0.24	0.78	0.309	0.309

Abbreviations: aMCI, amnestic mild cognitive impairment; S.E., standard error. ∗∗*p* < 0.01, ∗∗∗*p* < 0.001.

**Table 5:** Estimated fixed effects of predictors for approximate threshold of gap detection.

Fixed effects	Estimated coefficient (*β*)	S.E.	*t*	*p* value
Intercept	77.00	6.10	12.62	<0.001∗∗∗
Test ear				
Right	−1.75	8.63	−0.20	0.840
Left (reference)	0	0		
Group				
aMCI	−18.20	8.18	−2.22	0.029∗
Control (reference)	0	0		
Test ear∗ group	−6.05	11.57	−0.52	0.630

Abbreviations: aMCI, amnestic mild cognitive impairment; S.E., standard error. ∗*p* < 0.05, ∗∗∗*p* < 0.001.

## Data Availability

The data used to support the findings of this study are available from the corresponding author upon request.
